# Soliloquy of a Plastidule

**Published:** 1892-02

**Authors:** C. M. Wright


### Soliloquy of a Plastidule.

BY DR. C. M. WRIGHT, D.D.S.

Read before the Ohio State Dental Society.

I can't go back to the genesis of plastidules any easier than can that special organism known as man satisfactorily account for his origin, and the origin of his dwelling on the earth.

Man has studied the surface of the earth. and to a certain extent the position of this revolving ball in relation to other revolving bodies which he sees by reflected light all about him in the firmament.

Man has studied himself as far as he can. He has kept tolerably good records of his actions in various countries and times for a few thousands of years. He has been very curious about his own body too, and has devised some ingenious instruments to improve his eyesight, and then he has picked his relations to pieces after they have died, and made all sorts of experiments with the different pieces. He has stained and dried and moistened and teased and sliced and illuminated these pieces till he has been able to get down pretty close to bottom facts.

He has discovered protoplasm and for years has watched it in its motions and growth in other creatures.

He has discussed it and quarreled over it and guessed about it and has believed a great many things that he could not prove. These guesses he called hypotheses and builds his faith upon them. He has made some pretty shrewd guesses too, for he has guessed that we, the *molecules* of living matter exist, which is quite true, and he has even given us a name "Plastidule." We do not call ourselves 'Plastidules,' nor do we consider it a particularly attractive name, but when man makes such strenuous efforts to get acquainted with us; when he spends years in study to find us out; when he works so patiently with all his knowledge, accumulated mite by mite, for so many years in endeavors to see us as individuals, and acknowledges that he never expects to know more about us than can be gained from observations of what we do when congregated as squadrons, or entire communities, in masses which he calls proto-plasm, why, we naturally feel kindly toward man and do not quarrel with the name he has given us. We are to him part of the great *unknown*.

We are past finding out. We seem to occupy the same relation to man, as far as his perception goes, as does the God to whom he bows. Man studies God through what he calls the "handiwork of God," and he guesses about his "Creator," and forms images of Him in his mind of how He looks, and how He feels and thinks. He has no assurance that these images are exact—in fact he has every reason to believe that they are not, and yet he holds on to them and treasures them. He addresses prayers to them. He kneels down and supplicates, each man for himself, this god, a separate image of whom he has in his own mind, though he has never seen the original, and believes that his addresses will be listened to and in a measure answered.

Man can not see God but he thinks he knows that his God exists. Man can not see us—the plastidule—the molecule of protoplasm—made up possibly as man believes of still more minute and uncertain things which he calls atoms, and yet he thinks he knows that we exist, and he half suspects that we have very much to do with his past, present and future as a so-called organized being.

He does not worship us. It would look very ridiculous to us as well as to man if human beings should build churches with tall steeples and meet every now and then in these churches to offer up praise to us—the Plastidules.

Still we have the satisfaction of knowing that as men study about us more, as they spend more time guessing about us and thinking about us they have a very distinctly increased reverence for us. They reverence us as the Unknowable—the unknowable, though named, which so mysteriously envelops the life of man.

He can not define *life*, a word which he uses every day and thinks he knows about. He knows something about the manifestations of living beings and of living matter, and he strongly suspects that back of all these manifestations, these phenomena which he studies, is the plastidule. Well, so far, at least, he is right. There is a strong likeness existing between the life history of a man and the life history of a plastidule, between men and their relation to mother earth, and plastidules and their relation to mother man. The man had ancestors away back in ths dimmest past and during all that time, and now, and ever, he will be only a part of the earth the origin of which is said to be the heat of the sun. Every child born to man, whether he be at present of high or low degree in regard to health, or wealth or position, whether he is called king or whether he is called peasant, is an animal with a pedigree which we, the plastidule, can trace back so far into the dim past that even a description of the surface of the earth and of the condition of the atmosphere at that time can be but vaguely impressed upon the mind of the present man; a time when with difficulty we, the plastidules, rearranged the crude carbon and the gasses oxygen, hydrogen and nitrogen, the molecules of which are so unstable, into a diet for ourselves, so that we could exist as individuals and make up communities like the cell, and manifest a peculiar kind of force correlated to all force, and by slow and painful adaptation and re-arrangement of these same molecules and by constant effort toward perfection and harmonious action among ourselves we were enabled to unite as cells with special duties until we developed the organism which we now know as man.

It has taken us myriads of years to accomplish this, and yet we have remained the same and our work is going on. We are the unknown, the unknowable. Our laws of life govern all life. We exist and have always existed, for we are the *breath of life* that was breathed into the historic Adam. As the breath of life we inspire all so-called living matter whether in a one-celled micro-organism, a grain of corn, a majestic tree, a crawling worm, a buzzing insect, a chattering ape or a highly developed, intelligent man, and in this dominant creature man, this breath of life pervades the nerve cells in the cortex-cerebri making memory, imagination, reason, judgment, conscience possible to him just as it does the cells which by other modifications elaborate the shape and character of each individual's finger nails, and we are so intimately associated in each organism that the microscopic seed of that complex organism (which when properly planted is capable of producing another and similar complex organism) bears in its minute body the impress of every plastidule in the entire body, the impress, not only of the direct parent of the seed, but of past generations of parents and of all the possible crossings and variations and ramifications of past ancestral impressions. Man knows this fact and discusses the phenomena of atavism, of heredity, of a tendency to revert to a previous type. We are not only intimately associated, we plastidules, with every past and present impression made upon ourselves as we exist and have existed in the tissues high and low of an individual and his ancestors, but we are one with all other plastidules in the entire world of organisms and man is correlated to all not by blood, but by the breath of life.

Through us, the plastidule, man then is a part of all life, and the nearer he approaches harmonious correspondence the nearer will he fulfil his destiny.

